# A Study on the Profile of Poisoning in the Paediatric Population in a Tertiary Care Teaching Hospital of Chitradurga Region

**DOI:** 10.7759/cureus.32369

**Published:** 2022-12-09

**Authors:** Shreekrishna H K, Yatiraj Singi, Chandan V, Dipen Dabhi

**Affiliations:** 1 Forensic Medicine and Toxicology, Basaveshwara Medical College & Hospital, Chitradurga, IND; 2 Forensic Medicine and Toxicology, All India Institute of Medical Sciences, Bilaspur, IND; 3 Forensic Medicine, Akash Institute of Medical Sciences and Research Center, Devanahalli, IND

**Keywords:** paediatric population, socioeconomic status, kerosene, organophosphates, children, poisoning

## Abstract

Background

Most poisoning events among children are preventable and the major reason is lack of supervision by adults, including poor knowledge and attitude toward storage of such items. So, the prevention policy on children's poisoning shall take into account the age group, gender, socioeconomic status, residence, and other aspects such as the knowledge and attitude of adults. The present study was conducted to describe the profile of poisoning in the paediatric population in a tertiary care teaching hospital.

Methods

Our observational study was retrospective and was conducted at Basaveshwara Medical College and Hospital (BMCH), Chitradurga, Karnataka, under the Department of Forensic Medicine for a duration of three months (February 2021 to April 2021). Institutional ethical approval was obtained prior to the start of the study. As our study participants were paediatric patients (0-17 years) with acute poisoning (excluding homeopathic drug ingestion), a total of 81 paediatric patients' case sheets were finally reviewed and analysed. The data of paediatric poisoning cases were collected in a predesigned study proforma and included details about children’s age (in years), gender (male, female), residence (rural, urban), outcome (death, discharge), nature of poisoning (accidental, suicidal), and toxic agents in poisoning. The collected data were entered and analysed in the Microsoft Excel spreadsheet (Microsoft Corporation, Redmond, WA).

Results

The incidence of acute poisoning among the paediatric population in our teaching hospital was 1.4%. The most common age group with acute poisoning was 13-17 years (30.9%). The prevalence of acute poisoning was higher in male children (56.8%) when compared to female children (43.2%). Around three-fourths of paediatric cases (71.6%) with acute poisoning were having a rural residence. The overall mortality rate among children due to acute poisoning was 9.9%. The most common toxic agents involved in acute poisoning among children were organophosphate compounds (35.8%), followed by organochlorine compounds (30.9%) and pyrethrum compounds (11.1%).

Conclusion

From this study, we concluded that acute poisoning among children is mainly accidental, and the most common toxic agent responsible for the poisoning is pesticide or insecticide. Most poisoning events among children are preventable, and the major reason is a lack of supervision by adults, including poor knowledge and attitude toward storage of such items.

## Introduction

Poison is a substance that damages various human biological systems when absorbed through their epithelial surfaces. Among children, mechanical trauma (falls, injury from vehicles, or road traffic accidents) and poisoning constitute a significant proportion of mortality and morbidity. The incidence of poisoning is higher among toddlers and preschool children, which is due to their developmental phase [1]. With the mobility of infants along with exploration of surroundings and the tendency to put every object they rescue in their mouth, children, especially toddlers, are curious with an inability to judge between things that could be injurious or non-injurious to health, which makes them most susceptible to poisoning. Adding to this, the attractive visual appearance or packaging of poisonous items and the often tempting nature of those items also make the children (due to the motor development phase) prone to poisoning. With increasing age, children become particular in the selection of objects to be put in their mouth or for ingestion, and it decreases the rates of accidental poisoning with increased age [2].

In India, when compared to adults, poisoning among children is accidental instead of suicidal [3]. As a result of child poisoning, the annual bed occupancy rate in paediatric units ranges from 1% to 6%, out of which half of the children (3.9%) need admission to the paediatric intensive care unit (PICU). Adding to this, at present, when causes of children's hospitalization are observed, injury due to poisoning has evolved as the second most common cause. In recent times, a shift in the trend of poisoning among adolescents is seen, as suicidal poisoning is rising when compared to accidental poisoning, which is a matter of serious concern [4,5].

As compared to female children, male children are more prone to poisoning due to their higher active nature and explorative tendencies [6]. The storage of household items differs among people of various socioeconomic status (SES), as items are generally stored in open spaces on floors in houses with lower SES, and it makes it easy for children to access these items for exploring, which increases the incidence of poisoning among children with low SES as compared to higher SES [7]. Adding to this, in rural areas, agriculture is the main occupation and pesticides used for that purpose are not stored properly, which again makes it easy for children to access these pesticides. Also, people with lower SES and people residing in rural areas are still using kerosene as cooking fuel, which again becomes a potential source of poisoning when stored improperly [8-12]. The present study was conducted to describe the profile of poisoning in the paediatric population in a tertiary care teaching hospital.

## Materials and methods

Study design and subjects

Our observational study was retrospective and was conducted at Basaveshwara Medical College and Hospital (BMCH), Chitradurga, Karnataka, under the Department of Forensic Medicine for a duration of three months (February 2021 to April 2021) with the help of the Department of Paediatrics.

Institutional ethical approval was obtained from BMCH prior to the start of the study (approval number: BMCH/IEC/2020-2021/89; dated: 08/01/2021). During the defined period of study, the case sheets of paediatric patients with a confirmed history of poisoning in the last year (January 2020 to December 2020) were accessed and reviewed. As these were medicolegal cases (MLCs), so we, in addition, obtained the medical superintendent's permission prior to accessing and reviewing patient case sheets. Because our study participants were paediatric patients (0-17 years old) with acute poisoning (excluding homoeopathic drug ingestion), 81 paediatric patient case sheets were eventually reviewed and analysed.

Data collection

The data of paediatric poisoning cases were collected in a predesigned study proforma and included details about children’s age (in years), gender (male, female), residence (rural, urban), outcome (death, discharge), nature of poisoning (accidental, suicidal), and toxic agents in poisoning (organophosphates (OP) compounds, organochlorine (OC) compounds, pyrethrum compounds, kerosene, phenobarbitone, rat poison, paracetamol tablets and unknown tablets).

Statistical analysis

The collected data were entered and analysed in the Microsoft Excel Spreadsheet (Microsoft Corporation, Redmond, WA). The incidence rate of acute poisoning was estimated using the total number of acute poisonings among paediatric patients in a year as the numerator and total inpatients in the paediatric department during the same year as the denominator. Similarly, the mortality rate due to acute poisoning was calculated using paediatric mortality due to acute poisoning in a year as the numerator and the number of acute poisoning in paediatric cases in the same year as the denominator. Also, the mortality rates were calculated separately for male and female children.

## Results

In the present study, a total of 81 paediatric case sheets were reviewed. The hospital records also showed that there were a total of 5783 paediatric admissions in the year 2020. So, the incidence of acute poisoning among the paediatric population in our teaching hospital was 1.4%. In our study, the most common age group with acute poisoning was 13-17 years (30.9%), followed by three to five years (24.7%) (Table 1).

**Table 1:** Distribution of children with poisoning according to age (N = 81).

Age group	Frequency	%
<1 year	1	1.2
1-2 years	6	7.4
3-5 years	20	24.7
6-9 years	13	16.0
10-12 years	16	19.8
13-17 years	25	30.9

In our study, the prevalence of acute poisoning was higher in male children (56.8%) when compared to female children (43.2%). The difference in the occurrence of acute poisoning may be due to the tendency to explore and be more active in male children (Table 2).

**Table 2:** Distribution of children with poisoning according to gender (N = 81).

Gender	Frequency	%
Male	46	56.8
Female	35	43.2

In our study, around three-fourths of paediatric cases (71.6%) with acute poisoning were having a rural residence, whereas children from urban areas were 28.4%. Rural residents still use kerosene as their primary source of cooking fuel, which, if not stored properly, can become a potential source of poisoning (Table 3). In rural regions, agriculture is the main occupation, and pesticides are utilized for that purpose and again if not stored properly, can become a potential source of poisoning.

**Table 3:** Distribution of children with poisoning according to residence (N = 81).

Residence	Frequency	%
Rural	58	71.6
Urban	23	28.4

The mortality rate due to acute poisoning among male children was 10.9%, and it was 8.6% among female children. The overall mortality rate among children due to acute poisoning was 9.9% (Table 4).

**Table 4:** Mortality rate among children with poisoning (N = 81).

Gender	Deaths	Mortality rate
Male (n = 46)	5	10.9
Female (n = 35)	3	8.6
Overall (n = 81)	8	9.9

The nature of poisoning among more than two third of children (67.9%) was accidental, which clearly reflects the lack of supervision by adults, including poor knowledge and attitude toward the storage of such items. But suicidal poisoning among 30.9% of children is a matter of serious concern and points to the involvement of other departments (social welfare) for the prevention of such a portion of suicidal poisoning. In our study, one case of homicidal poisoning was also observed (Table 5).

**Table 5:** Nature of poisoning among children (N = 81).

Nature of poisoning	Frequency	%
Accidental	55	67.9
Suicidal	25	30.9
Homicidal	1	1.2

The most common toxic agents involved in acute poisoning among children were organophosphates (OP) compounds (35.8%), followed by organochlorine (OC) compounds (30.9%) and pyrethrum compounds (11.1%), which is quite obvious as three-fourth of paediatric acute poisoning cases were from rural areas (pesticide usage in agriculture) in our study. Also, acute poisoning due to prescription drugs was seen in more than one-tenth of paediatric cases (phenobarbitone (4.9%) and paracetamol (6.2%)). Consumption of unknown tablets in 6.2% of children shows the lack of supervision by adults, which further complicates the poisoning cases due to the nonspecific nature of treatment for unknown drugs or tablets (Table 6).

**Table 6:** Toxic agents involved in children with poisoning (N = 81).

Toxic agent	Frequency	%
Organophosphates (OP) compounds	29	35.8
Organochlorine (OC) compounds	25	30.9
Pyrethrum compounds	9	11.1
Kerosene	1	1.2
Phenobarbitone	4	4.9
Rat poison	3	3.7
Paracetamol tablets	5	6.2
Unknown tablets	5	6.2

## Discussion

In our study, we have described the profile of acute poisoning in the paediatric population admitted to a tertiary care teaching hospital. Whether it is in India or across the world, paediatric acute poisoning is one of the most preventable conditions, which can reduce mortality and morbidity among children to a significant number. In India, as a result of child poisoning, the annual bed occupancy rate in paediatric units of various hospitals ranges from 0.3% to 7.6%, out of which half of the children need admission to PICU [13,14]. In our study, the incidence of acute poisoning among the paediatric population in the present teaching hospital was 1.4%. Similar to our study, studies by Aggarwal et al. and Manikyamba et al. showed the incidence of acute poisoning among the paediatric population in tertiary care centres as 2.1% and 2.8%, respectively [9,15]. Also, the proportion of children who needed PICU admission was similar to the studies by Reddy et al. and Selvakumar et al. [16,17].

In our study, the prevalence of acute poisoning was higher in male children (56.8%) when compared to female children (43.2%). Similarly, studies by Kohli et al., Ram et al., Polasa et al., Gupta et al., and Singh et al. have shown the prevalence of acute poisoning is higher among males as compared to females [8,18-21].

In our study, the most common age group with acute poisoning was 13-17 years (30.9%), followed by three to five years (24.7%). In our study, one-third of acute poisoning cases (33.3%) occurred in children aged zero to five years, which was incoherent with studies by Dutta et al., Polasa et al., Gupta et al., Gummin et al., and Ghosh et al., where the majority of acute poisoning cases occurred in children less than six years of age [14,19,20,22,23].

In our study, the nature of poisoning among more than two-thirds of children (67.9%) was accidental, which was similar to the studies done by Kohli et al., Polasa et al., Gupta et al., Gummin et al., Ghosh et al., and Yadav et al. [8,19,20,22-24], where the majority of cases were accidental and it demonstrates a lack of supervision by adults, including a lack of understanding and attitude toward storage of such items, as well as exploration of surroundings and a tendency for children to put every object rescued in their mouths.

In our study, the most common toxic agents involved in acute poisoning among children were organophosphates (OP) compounds (35.8%), followed by organochlorine (OC) compounds (30.9%) and pyrethrum compounds (11.1%), which is quite obvious, as three-fourth of paediatric acute poisoning cases were from rural areas (pesticide usage in agriculture) in our study. A similar observation was found in studies by Bhat et al., Budhathoki et al., and Mandal et al., where the most common cause of paediatric poisoning was either pesticides or insecticides [11,25,26]. In our study, acute poisoning due to kerosene was observed among one child, which was in contrast to the studies by Aggarwal et al., Dutta et al., Polasa et al., and Ghosh et al., where a number of cases of poisoning were due to kerosene [9,14,19,23].

In our study, acute poisoning due to prescription drugs was seen in more than one-tenth of paediatric cases (phenobarbitone (4.9%) and paracetamol (6.2%)). In a study by Roy et al., medicinal drugs (thyroxine, phenytoin, and benzodiazepines) were the most common cause of acute poisoning among children [6]. In a study by Manikyamba et al., the most common medicinal drugs causing acute poisoning were thyroxine, phenytoin, benzodiazepines, paracetamol, cyproheptadine, Zandu Balm, telmisartan, and carbamazepine [15].

In our study, the overall mortality rate among children due to acute poisoning was 9.9%. Similar mortality rates among children due to acute poisoning were observed in studies by Mandal et al. and Modi et al. [25,27].

Limitation

The limitation of the study is that we should have compiled the hospital records of at least the last five years to note a comparative trend of acute poisoning among children.

## Conclusions

From this study, we concluded that acute poisoning among children is mainly of accidental nature and the most common toxic agent responsible for the poisoning is pesticides or insecticides. Most poisoning events among children are preventable and the major reason is lack of supervision by adults, including poor knowledge and attitude toward storage of such items. So, the prevention policy on children's poisoning shall take into account the age group, gender, SES, residence, and other aspects such as the knowledge and attitude of adults.
